# Idiopathic Granulomatous Inflammation of the Periocular Structures in Four Dogs: Clinical, Histopathological, and Imaging Findings and Therapeutic Outcome

**DOI:** 10.1111/vop.70275

**Published:** 2026-09-26

**Authors:** J. Kurwinkel, C. Busse, A. Beineke, I. Gerhauser, S. Pahl, A. H. Marx

**Affiliations:** ^1^ Department of Small Animal Medicine and Surgery University of Veterinary Medicine Hannover, Foundation Hannover Germany; ^2^ Department of Pathology University of Veterinary Medicine Hannover, Foundation Hannover Germany

**Keywords:** granulomatous inflammation, idiopathic, mass lesion, periocular structures, steroid responsive

## Abstract

**Purpose:**

To describe cases of canine idiopathic periocular granulomatous inflammation.

**Material and Methods:**

Clinical records were reviewed.

**Animal Studied:**

At the time of presentation, the four patients (German Shepherd dog, mixed breed, Magyar Vizsla, Hovawart) were 1.5–5 (mean 3.75) years old. All dogs were female; two were spayed. Lesions were unilateral in 3/4 cases. Dogs presented with ulcerated (1) or non‐ulcerated (3) mass lesions affecting the periocular skin (3/4), medial canthal area (2/4), or third eyelid (1/4).

**Results:**

Incisional biopsies revealed pyogranulomatous inflammation in three and granulomatous inflammation in one case. There was no evidence of microbial infection in the specimens. Patients received anti‐inflammatory or immunosuppressive dosages of systemic corticosteroids. Three dogs also received systemic cyclosporin (5 mg/kg, sid), and one dog received topical cyclosporin (0.1%, bid). One dog underwent surgical debulking of a circumscribed mass within the left lower eyelid prior to immunosuppressive therapy and developed an additional swelling on the left upper eyelid. Remission of clinical signs was achieved in all patients. Dogs were treated for 5 to 11 months (mean 8.7). No recurrence was reported in 4/4 patients during a mean follow‐up period of 17.3 months. In 3/4patients, there was no recurrence within an average of 11 months after discontinuation of therapy. One dog was lost to follow‐up.

**Conclusion:**

Idiopathic granulomatous inflammation is a differential diagnosis for periocular mass lesions. Its diagnosis is based on histopathology and the exclusion of other causes of granulomatous inflammation.

## 1 Introduction

Granulomatous inflammation represents a chronic immune response typically characterized by aggregation of macrophages, epithelioid cells, and multinucleated giant cells. It occurs as a reaction to persistent antigenic stimulation [1]. The etiology of granulomatous inflammation can be categorized into infectious, neoplastic, autoimmune, allergic, toxic, and idiopathic conditions [1, 2, 3, 4]. Pyogranulomatous inflammation is characterized by the predominance of macrophages and neutrophilic granulocytes, often accompanied by plasma and giant cells [5]. Histopathological identification of this type of inflammation can narrow down the list of differential diagnoses to infectious, immune‐mediated, and idiopathic diseases [6, 7].

Sterile forms of pyogranulomatous inflammation affecting ocular structures have been described in dogs. Sterile pyogranulomatous blepharitis is a presumed immune‐mediated condition affecting dogs and is diagnosed based on clinical presentation, histopathology, and by ruling out infectious diseases [1, 6]. Inflammation is usually confined to the eyelid tissue and does not extend further [6, 8] (REF). Solitary forms of granulomatous inflammation can also occur, such as in the case of a meibomian gland rupture, leading to the release of sebum into the eyelid tissue and therefore causing an acute foreign body reaction [8]. Over time, this reaction can become chronic and form soft tissue masses (chalazion, pyogranulomas) [6, 7].

Here, we described atypical periocular granulomatous lesions in four dogs that presented as mass lesions that not only involved eyelid tissue, as typically seen in cases with sterile pyogranulomatous blepharitis, but extended into the periocular skin and adjacent ocular structures within the orbit, clinically raising the concern of a malignant process.

## 2 Case Presentations

The patient data, reported clinical signs, and previous treatments are summarized in Table 1. None of the patients responded to initial therapy.

**TABLE 1:** Signalment, anamnesis, clinical and histopathological presentation and treatment are shown. OD: Right eye, OS: Left eye, CsA: Cyclosporine, Pred: Prednisolone.

	Case 1	Case 2	Case 3	Case 4
**Breed**	German Shepherd	Mixed breed	Magyar Vizsla	Hovawart
**Age**	5 years	1.5 years	4.5 years	4 years
**Sex**	female, spayed	female, spayed	female	female
**Travel history**	none	Greece	none	none
**Vaccination**	yes	yes	yes	NA
**Deworming**	yes	yes	yes	NA
**Concurrent diseases**	none	none	none	none
**Duration of clinical signs prior to presentation**	3 weeks	3 weeks	8 weeks	4 weeks
**Anamnesis**	**OS** upper eyelid swelling and hyperemia, mucous discharge, signs of discomfort (scratch in affected side)	**OS** conjunctival hyperemia, progressive protrusion of nictitating membrane **OD** conjunctival hyperemia	**OD** periocular swelling, stye on upper eyelid, progressive swelling of lateral canthus extending to upper eyelid	**OS** serosanguineous discharge from left upper eyelid, reported ocular inflammation
**Previous Treatment**	Topical: unknown antibiotic, CsA Systemic: unknown antibiotic	Topical: neomycin sulfate, hydrocortisone acetate, lidocaine hydrochloride q 12/h, switched to prednisolone acetate	Topical: unknown antibiotic Systemic: initially unknown NSAID, then switched to prednisolone 1 mg/kg SID	Topical: unknown antibiotic Systemic: unknown antibiotic and carprofen
**General examination**	unremarkable	bilateral moderately enlarged mandibular, popliteal, axillary, and cranial cervical lymph nodes	unremarkable	unremarkable
**Clinical presentation**	**OS:** Moderate serous discharge; hyperemic, ulcerated mass in the nasal aspect of the upper eyelid and medial canthus, affecting the eyelid margin, palpebral conjunctiva, and surrounding skin. The eyelid structure is no longer discernible due to the infiltrative changes nature of the mass.	**OS:** Severe nodular swelling of the nictitating membrane; moderate enophthalmos; Firm, nodular swelling on the temporal side of the left lower eyelid, with slight swelling of the upper eyelid. **OD:** Moderate conjunctival hyperemia; follicles in the bulbar conjunctiva of the nictitating membrane; slight chemosis.	**OD:** diffuse, poorly demarcated, hyperemic swelling along the upper eyelid above the meibomian glands; crusty surface, releasing discharge upon manipulation; mild hyperemia of the palpebral conjunctiva.	**OS:** mild sero‐sanguinous discharge; palpebral conjunctival hyperemia and chemosis; non‐ulcerative mass lesion (approx. 10 × 6 mm) in the lower eyelid. The lower lacrimal punctum could not be cannulated. **OD:** Mild palpebral conjunctival hyperemia.
**Blood analysis (hematology, blood chemistry)**	unremarkable	unremarkable	unremarkable	unremarkable
**Biopsy**	Incisional biopsy size 4 mm	Incisional biopsy size 4 mm	Incisional biopsy size 4 mm	Incisional biopsy size 4 mm and excisional biopsy
**Histopathology**	pyogranulomatous inflammation with granulation tissue	granulomatous inflammation	pyogranulomatous inflammation	pyogranulomatous inflammation with granulation tissue
**Ziehl‐Neelsen Staining**	negative	negative	negative	negative
**Periodic acid‐Schiff reaction**	—	negative	negative	—
**Treatment**	medical (systemic: Pred, CsA)	medical (topical: CsA, systemic: meloxicam initially, later Pred)	medical (systemic: Pred, CsA)	medical & surgical (topical: neomycin sulfate, polymyxin B sulfate, dexamethasone, systemic: Pred, CsA)
**Follow‐up since treatment started**	19 months	18 months	20 months	12 months
**Follow‐up after discontinuation of therapy**	8 months	14 months	10 months	7 months, until the patient was lost to follow‐up.
**Reoccurrence**	no	no	no	no

### 2.1 Case 1

**FIGURE 1:**
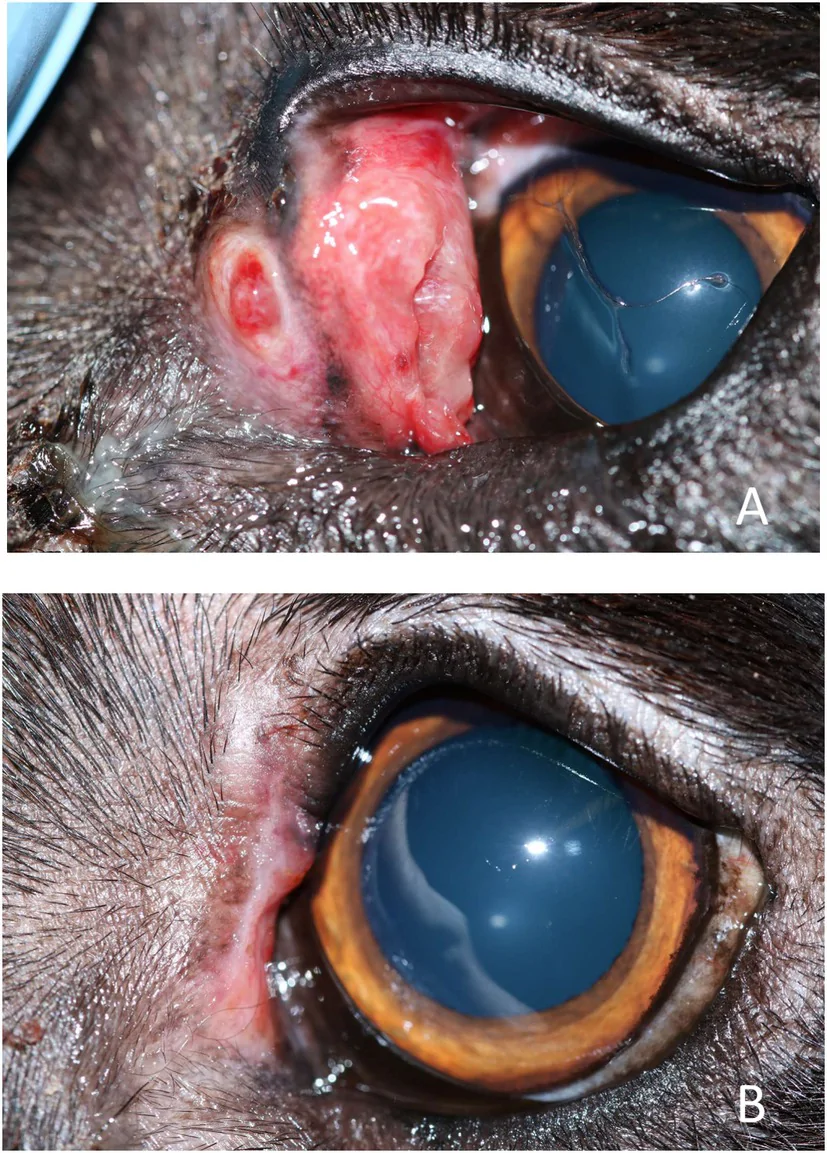
(A) Left eye of patient 1 showing a hyperemic, ulcerated mass located in the nasal aspect of the upper eyelid and medial canthus, affecting the eyelid margin, palpebral conjunctiva, and surrounding skin. (B) Presentation 11 months after initiation of therapy, the lesion has completely resolved, leaving behind only mild fibrosis within the medial canthal area.

On ophthalmic examination, the left eye demonstrated moderate serous discharge and a hyperemic, ulcerated mass located in the nasal aspect of the upper eyelid and medial canthus, affecting the eyelid margin, palpebral conjunctiva, and surrounding skin (Figure 1A). The eyelid structure in this region was no longer discernible due to the infiltrative nature of the mass. The remainder of the ophthalmic examination was unremarkable.

**FIGURE 2:**
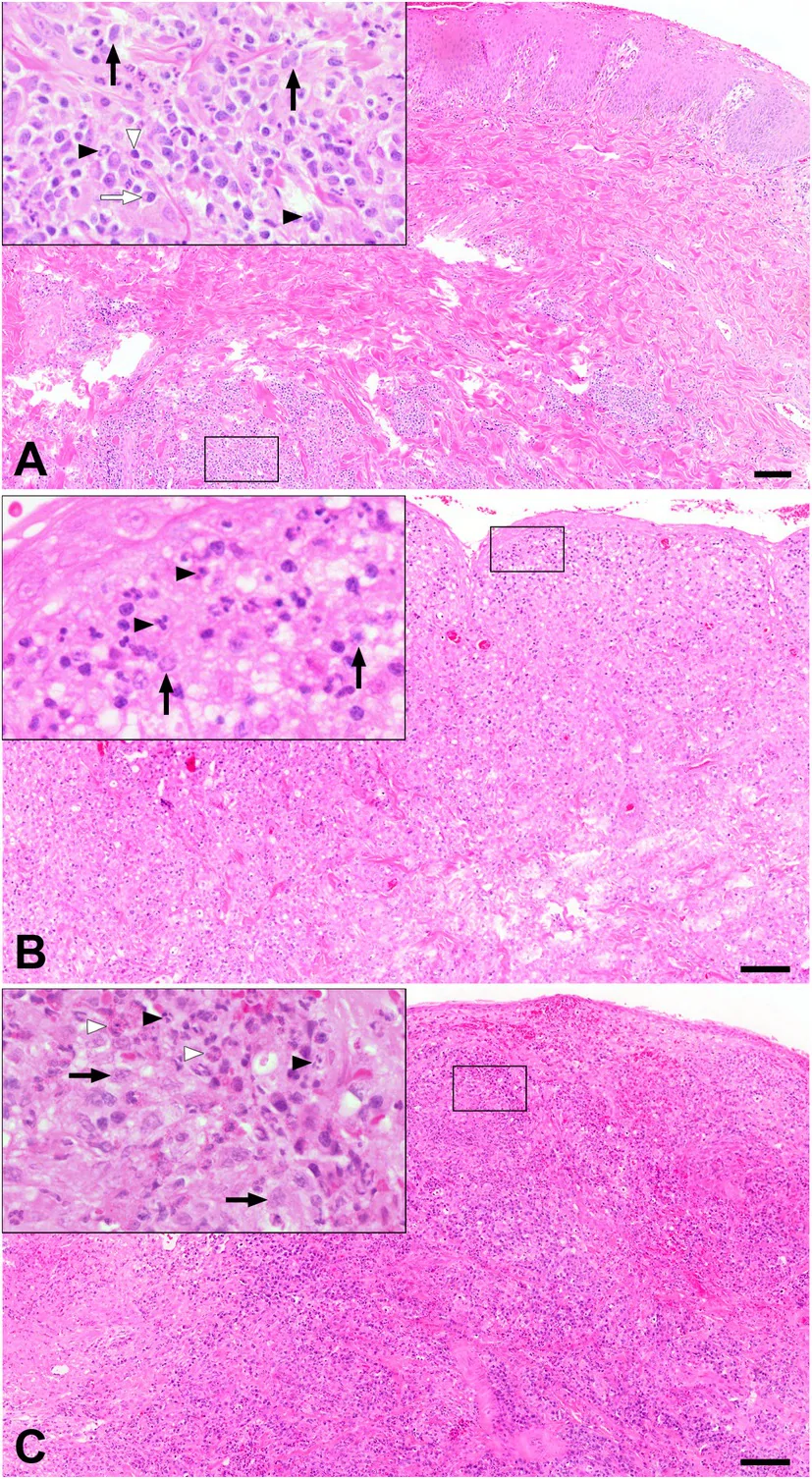
Histopathological lesions in patient 1 (A), patient 2 (B), and patient 4 (C). Multifocal to diffuse pyogranulomatous inflammation characterized by infiltration of neutrophils (black arrowheads) and macrophages (black arrows). Note single lymphocytes (white arrowhead) and plasma cells (white arrow) in patient 1 (A) and additional eosinophils (white arrowheads) in the inflammatory lesions of patient 4 (C). Insets show higher magnifications of the areas indicated by the black rectangles. Bars = 100 μm.

An incisional biopsy of the medial canthus was performed, revealing an ulcerative and pyogranulomatous inflammation characterized by significant infiltration of macrophages and neutrophils and lower numbers of plasma cells, lymphocytes, and eosinophils, which was associated with mild granulation tissue formation (Figure 2A). Ziehl‐Neelsen staining was negative for acid‐fast organisms.

Following biopsy results, oral prednisolone (PredniTab, CP Pharma, Germany) therapy was initiated at 1 mg/kg SID for 1 week, during which progression to bilateral disease was seen, and the dosage was increased to 2 mg/kg SID. The dosage was reduced to 1.5 mg/kg SID 4 days later due to clinical improvement and the development of side effects (polydipsia, polyuria, polyphagia) attributable to systemic corticosteroid therapy. The medication was continued for 10 weeks, resulting in a continuously decreasing size of the mass lesion and increased patient comfort. Oral cyclosporine (Sporimmune 50 mg/mL, Dechra Veterinary Products Deutschland GmbH, Germany) 5 mg/kg SID was added to the treatment regimen, and prednisolone was tapered off over the next 12 weeks, during which the lesions completely resolved, leaving behind only a mild fibrosis within the medial canthus (Figure 1B). After discontinuation of prednisolone, cyclosporine (Sporimmune 50 mg/mL, Dechra Veterinary Products Deutschland GmbH, Germany) was gradually tapered and was completely discontinued 11 months after diagnosis. There was no relapse for 8 months after discontinuing therapy.

### 2.2 Case 2

**FIGURE 3:**
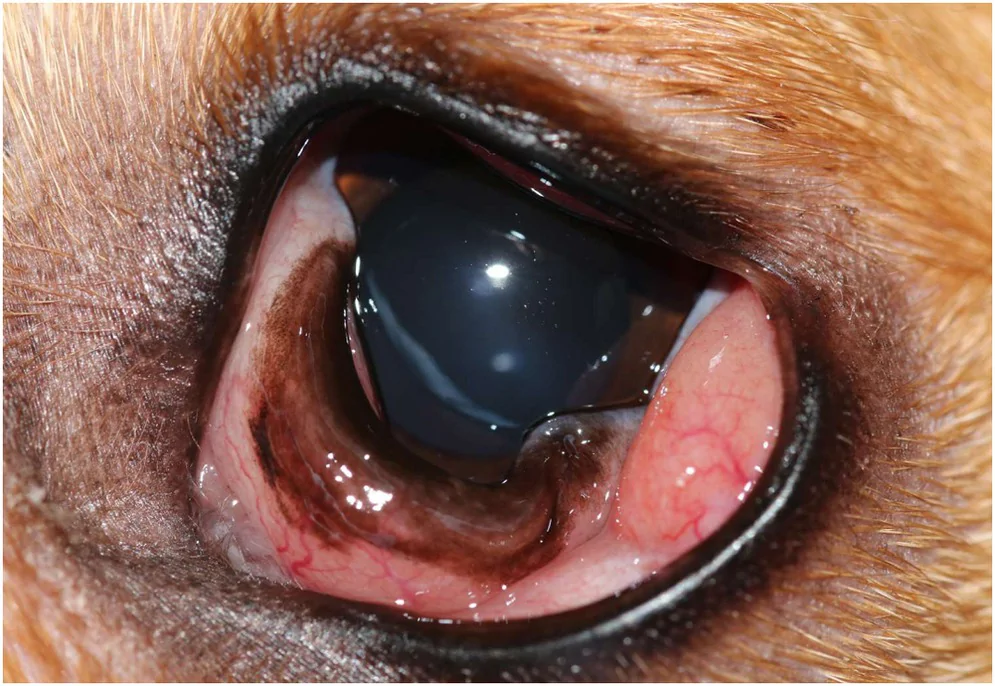
A left eye of patient 2 with swelling of the nictitating membrane and firm swelling in the temporal aspect of the left lower eyelid, with slight swelling of the upper eyelid.

The left eye showed a large nodular swelling of the nictitating membrane, resulting in moderate enophthalmos. There was a well‐circumscribed nodular and firm swelling in the temporal aspect of the left lower eyelid, with slight swelling of the upper eyelid. (Figure 3). The right eye presented with moderate conjunctival hyperemia and follicles in the bulbar conjunctiva of the nictitating membrane, with slight chemosis. The remainder of the ophthalmic examination was within normal limits.

The patient was sedated for incisional biopsy sampling of the swelling in the left lower eyelid and the third eyelid and fine needle aspiration cytology of the right mandibular (cranial portion: 3.0 cm; caudal portion: 1.5 cm; contralateral left cervical lymph node: 1.2 cm) and left popliteal lymph nodes (1.5 cm; contralateral right popliteal lymph node: 1.0 cm), given the suspicion of a potentially malignant neoplasia. Histopathology revealed diffuse subepithelial infiltration with macrophages, lymphocytes, and a few neutrophils consistent with diffuse severe chronic granulomatous inflammation (Figure 2B). There was no evidence of bacterial (Ziehl‐Neelsen staining: negative), mycotic (Periodic acid‐Schiff reaction: negative), or protozoal infection. Fine needle aspiration cytology of the lymph nodes revealed medium‐sized lymphocytes (60%–70%), blastoid cells (10%–20%), few plasma cells, macrophages, neutrophil granulocytes, and one mitotic figure per field of view, consistent with a reactive hyperplastic process.

Serology was performed and included *Neospora, Babesia canis, Ehrlichia canis, Leishmania, and Rickettsia conori*. PCR was performed and included *Dirofilaria, Filaria, Piroplasma, Hepatozoon*, and *Anaplasma platys* . A borderline elevated *Ehrlichia canis* antibody titer was present; the remaining tests were negative. Initial therapy consisted of oral meloxicam (Metacam, Boehringer Ingelheim Vetmedica, Germany) at 0.1 mg/kg SID for 5 days. Upon receiving histopathology results, therapy was switched to oral prednisolone (PredniTab, CP Pharma, Germany), 1 mg/kg SID. After 2 weeks, there was a significant improvement. The conjunctival hyperemia of the nictitating membrane and the chemosis in the left eye were less prominent, and follicles resolved. The left eye showed only mild remaining enophthalmos, and the well‐circumscribed swelling in the temporal aspect of the lower and upper eyelids was significantly smaller. The patient showed polyuria and polydipsia consistent with systemic side effects of corticosteroid therapy but was otherwise in good condition. The therapy was continued with 1 mg/kg prednisolone SID, and topical cyclosporine 0.1% (Ikervis, Santen, Switzerland) was added, one drop in each eye BID. After 4 weeks, symptoms had almost completely subsided, and prednisolone tapering began. Further medical management of the patient was taken over by the referring veterinarian, who reduced the prednisolone and topical cyclosporine and discontinued them 5 months after the initial diagnosis. There was no relapse 14 months after discontinuing therapy.

### 2.3 Case 3

**FIGURE 4:**
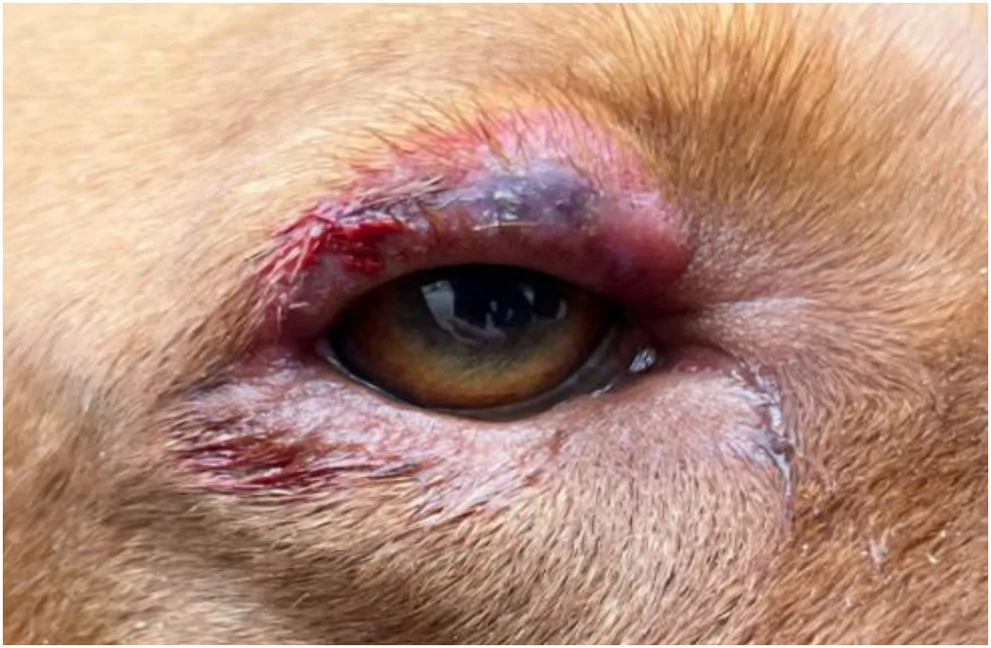
The right eye of patient 3 showing a diffuse, poorly demarcated swelling and a crusty surface along the upper eyelid above the level of the meibomian glands.

The right eye showed a diffuse, poorly demarcated hyperemic swelling in the area of the upper eyelid with crusty deposits in the central third; upon slight pressure, macroscopically purulent discharge was released (Figure 4). Mild hyperemia of the palpebral conjunctiva was noted. The remainder of the ophthalmic examination was within normal limits.

Cytological diagnosis of the discharge revealed purulent inflammation with occasional epithelial cells. To rule out a neoplastic process, an incisional biopsy was performed for histopathological examination. During sampling, the skin appeared very thin and easily torn when manipulated, leaving a half‐moon‐shaped area of ulceration above the upper eyelid. Simple interrupted sutures using 6–0 polyglactin 910 (Vicryl) were used to close the wound.

Histopathological analysis revealed moderate multifocal perivascular infiltration of the superficial dermis with macrophages and neutrophils, with single lymphocytes and plasma cells. The final diagnosis was moderate, multifocal, perivascular pyogranulomatous superficial dermatitis. Ziehl‐Neelsen staining for acid‐fast bacteria, Periodic acid‐Schiff reaction for fungal structures, and serology for leishmaniasis antibodies were negative.

Based on these findings, a treatment regimen of prednisolone (Prednitab, CP Pharma, Germany) 1 mg/kg SID was initiated. At the onset of therapy, similar lesions had appeared in the nasal aspect of the upper eyelid. At the 7‐day follow‐up, the swelling in the right eye had significantly subsided, and the biopsy site healed. The patient developed polyuria and polydipsia and gained about 18% body weight during the course of treatment. Oral cyclosporine (Sporimmune 50 mg/mL, Dechra Veterinary Products Deutschland GmbH, Germany) 5 mg/kg SID was added to the therapy to allow for decreasing the corticosteroid dose to reduce side effects. Tapering of prednisolone began 2 weeks later over a course of 14 weeks. Subsequently, cyclosporine was also tapered (3 mg/kg) under frequent monitoring, over a course of approximately 5 months. The follow‐up period lasted 20 months, with no recurrence observed even after therapy was discontinued at the end of a 10‐month treatment period.

### 2.4 Case 4

**FIGURE 5:**
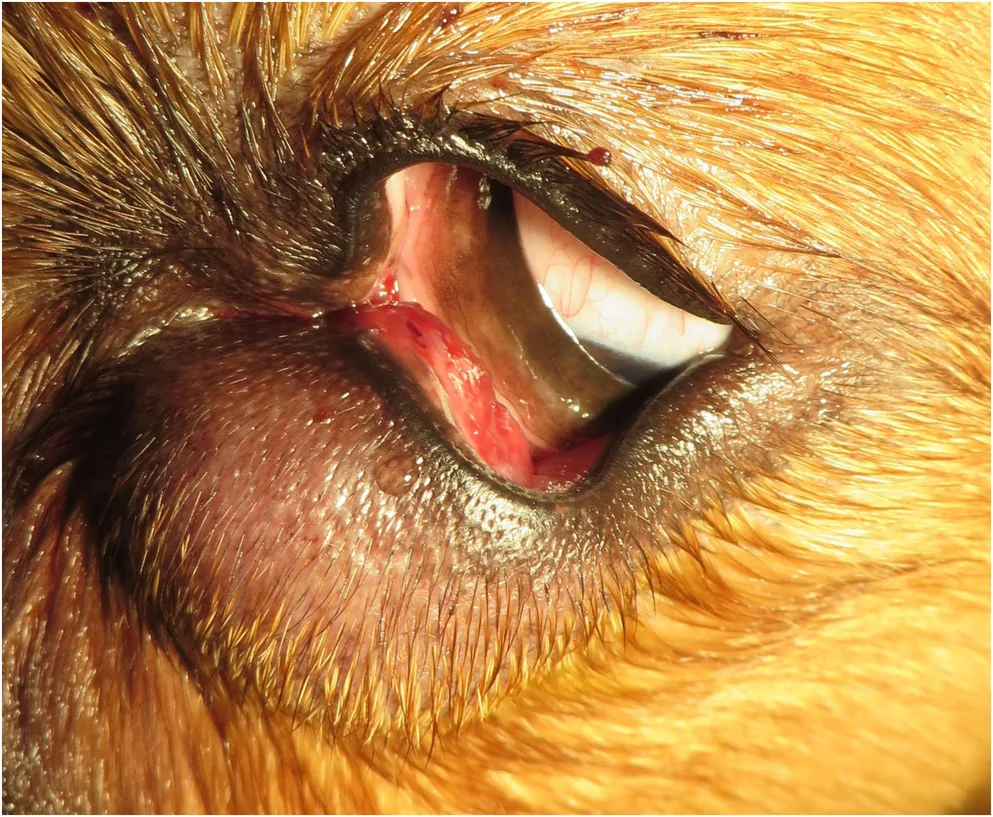
The left eye of patient 4 showing sero‐sanguinous discharge; palpebral conjunctival hyperemia and chemosis; non‐ulcerative mass lesion measuring approximately 10 × 6 mm in the lower eyelid.

Ophthalmic examination revealed a non‐ulcerative mass lesion measuring approximately 10 × 6 mm within the left lower eyelid, mild serosanguinous discharge, palpebral conjunctival hyperemia, and chemosis (Figure 5). The lower lacrimal punctum of the left eye could not be cannulated. The right eye showed mild palpebral conjunctival hyperemia. The remainder of the ophthalmic examination was unremarkable. The microbiological analysis of the discharge revealed no bacterial growth. An incisional biopsy (4 mm) of the left lower eyelid was obtained under sedation. Severe diffuse pyogranulomatous inflammation was predominantly characterized by neutrophils and macrophages, with occasional eosinophilic granulocytes present (Figure 2C).

Pending microbiological and histopathological results, empirical therapy was initiated, consisting of meloxicam 0,1 mg/kg SID (Melosus CP‐ Pharma Handelsgesellschaft mbh, Germany), metronidazole 10 mg/kg TID (Metrobactin 250 mg, Dechra Veterinary Products Deutschland GmbH, Germany), and topical chloramphenicol (Posifenicol 1% URSAPHARM Arzneimittel GmbH, Germany) in both eyes four times daily. After 7 days of therapy, no clinical improvement was observed. Consequently, the mass lesion was surgically removed under general anesthesia. Histopathological examination of the excised tissue confirmed pyogranulomatous inflammation with granulation tissue formation, predominantly characterized by neutrophils and macrophages, with occasional eosinophilic granulocytes present. Ziehl‐Neelsen staining was negative for acid‐fast bacteria.

After removal of the mass lesion via a cutaneous incision parallel to the eyelid margin with excisional resection in two separate specimens measuring approximately 2 × 2 cm, while sparing the eyelid margin and lacrimal puncta, a swelling began to develop in the upper eyelid; wound closure was performed using interrupted 6–0 polydioxanone (PDS Plus Ethicon Germany) for subcutaneous and Polyamid 4–0 (Ethilon Ethicon Germany) for skin sutures. Upon receiving histopathology results, therapy was adjusted: meloxicam, systemic antibiotics, and topical chloramphenicol were discontinued, and oral prednisolone (PredniTab CP‐ Pharma Handelsgesellschaft mbh, Germany) 0.8 mg/kg SID was initiated. Additionally, topical therapy with dexamethasone, neomycin sulfate, and polymyxin B sulfate (Isopto‐Max, Novartis Pharma GmbH, Germany) was prescribed. Fourteen days after the initial presentation, the left eye showed marked reduction in eyelid swelling. The topical therapy was discontinued. The contralateral eye developed temporal perilimbal corneal edema and neovascularization, signs consistent with episcleritis, which resolved with dexamethasone eye drops (Dexavet, CP‐Pharma Handelsgesellschaft mbH, Germany), administered BID in both eyes. Due to the development of gastroenteritis, systemic prednisolone therapy was temporarily discontinued, resulting in an immediate relapse of symptoms in the left eye. The episcleritis in the right eye was managed with topical prednisolone acetate (Inflanefran, AbbVie Deutschland GmbH&CoKG, Germany) administered TID and cyclosporine 0,1% (Optimmune Merck&Co. Inc., Rathway, NJ, USA) ointment administered BID. Over the course of 4 months, symptoms improved, and the mass lesion of the left eye did not return, and therapy was adjusted to include oral cyclosporine (Sporimmune, Dechra Veterinary Products Deutschland GmbH, Germany) 5 mg/kg SID, while prednisolone was tapered off and discontinued after 5 months. There was no relapse 7 months after discontinuing therapy until the patient was lost to follow‐up.

## 3 Discussion

This case series describes canine patients presenting with granulomatous lesions affecting periocular tissues. In all cases, the presence of fungi, acid‐fast bacteria, or algae was further investigated using routine Hematoxylin & Eosin staining (H.E.) in all cases, Periodic acid‐Schiff reaction in two cases, and Ziehl‐Neelsen staining in all cases. Serology to identify the presence of antibodies against *Leishmania infantum* was performed in Case two, a dog that had a travel history to an endemic area. The remaining cases lacked travel history, which made Leishmaniasis an unlikely diagnosis. As no underlying infectious etiology or other etiology could be identified based on the diagnostic investigations performed, the granulomatous lesions in these cases were considered idiopathic.

Idiopathic pyogranulomatous inflammation was reported in various ocular structures in dogs, including the eyelids and the orbit. Cases involving the eyelids were characterized by localized granulomatous/pyogranulomatous inflammation of the eyelid margins with predominant macrophage and neutrophilic infiltration [6, 7]. Idiopathic orbital inflammation, previously termed orbital pseudotumor, a well‐described condition in human medicine, encompasses a heterogeneous group of disorders characterized by non‐specific orbital signs of inflammation, with no identifiable local or systemic cause [9]. Histologically, it is defined by sterile pyogranulomatous inflammation, featuring infiltration of mainly macrophages and neutrophils and lower numbers of lymphocytes, plasma cells, and eosinophils [10]. None of the lesions described in our cases were confined to the eyelid margin as described previously [6]. In this study, lesions extended into the medial aspect of the orbit in one case, involved deep eyelid structures or areas of periocular skin reaching beyond the level of meibomian glands in three cases, and involved the nictitating membrane in one case. Histological features of our cases were consistent with those of pyogranulomatous blepharitis and idiopathic orbital inflammation, being pyogranulomatous in three cases and granulomatous in one case.

Typical clinical features of idiopathic orbital inflammation in humans include orbital pain and the presence of a diffuse or delimited space‐occupying lesion, which can occur acutely. The eyelids may also be affected in 1%–22% of cases [11, 12]. Diagnosis in human medicine is based on magnetic resonance imaging or computed tomography findings, alongside clinical examination, thyroid function tests (T4 and TSH), and histopathology [13, 14]. Idiopathic orbital inflammation is typically unilateral, with bilateral involvement being rare [9, 13]. The condition is usually steroid‐responsive and can present in acute or chronic forms [13]. In veterinary medicine, a similar condition was described in a bull terrier in 1998, where a red mass protruded from the lacrimal punctum [15], which was also seen in case four. A recently published case series by Freitas et al. [16] described four dogs affected by similar symptoms and established the term ‘canine idiopathic orbital inflammation’ [16]. In our study, space‐occupying orbital lesions were observed in all cases, presenting with enophthalmos in one case. Given the similarity of histopathological findings and response to therapy, we speculate that our cases have the same underlying etiology as idiopathic orbital inflammation in humans and dogs and/or represent the same condition in a different location.

As previously stated, the diagnosis of idiopathic orbital inflammation is established primarily through the exclusion of infectious causes. In the present study, histopathological examination, including hematoxylin and eosin, Ziehl–Neelsen staining, and PAS staining, did not reveal infectious organisms in any patient [16]. Serological testing was performed in only one case, while the remaining dogs had no history of travel to regions endemic for *Leishmania* [3].

Treatment of idiopathic orbital inflammation in humans consists of systemic corticosteroids, which often alleviate the symptoms [14] using dosages of 1–1.5 mg/kg prednisolone once daily for 1 to 2 weeks. After this time, prednisolone is phased out over 5 to 8 weeks. A retrospective study in human medicine describes symptom relief in 63% of cases with idiopathic orbital inflammation [17]. Some studies showed that intraorbital or periorbital injection of triamcinolone acetonide 20–40 mg/injection is an effective method to treat idiopathic orbital inflammation, and the injection could be repeated every 4 weeks [18, 19, 20]. If corticosteroids are not sufficient, the second treatment option in humans consists of low‐dose radiotherapy or the administration of immunosuppressants such as cyclosporin, cyclophosphamide, methotrexate, azathioprine, or indomethacin [21]. In the case series of Freitas et al. [16], one case was treated surgically by exenteration, and the lesions recurred within a month [16]. In our study, all cases were treated with either anti‐inflammatory or immunosuppressive dosages of systemic corticosteroids (1–2 mg/kg/day), while one case also underwent surgical debulking. Cyclosporin was administered systemically in three cases at a dosage of 5 mg/kg/day and Cyclosporin 0,1% topically in one case. In all cases, therapy was discontinued after 5–11 months. Based on the available information, no recurrences were reported after the completion of therapy with a follow‐up of 12–20 (mean 17.3) months.

## 4 Conclusion

Idiopathic granulomatous/pyogranulomatous inflammation should be considered as a differential diagnosis for mass lesions affecting periocular structures with or without extension into the orbit. The diagnosis is based on histopathological examination and the exclusion of other causes for granulomatous/pyogranulomatous inflammation. Response to immunosuppressive therapy was good in all cases described in this study, resulting in complete resolution of the disease.

## Author Contributions

**J. Kurwinkel:** writing – review and editing, writing – original draft, methodology, validation, visualization, conceptualization. **C. Busse:** supervision. **A. Beineke:** investigation. **I. Gerhauser:** investigation, writing – review and editing. **S. Pahl:** visualization. **A. H. Marx:** project administration, writing – review and editing, methodology, conceptualization, investigation, supervision.

## Disclosure

Artificial Intelligence: The authors have not used AI to generate any part of the manuscript.

## Ethics Statement

Animal owners or owners' representatives provided written consent for the treatment provided and for the publication of data and images.

## Conflicts of Interest

The authors declare no conflicts of interest.

## Data Availability

The data that support the findings of this study are available from the corresponding author upon reasonable request.
